# Integration of DSRC, mmWave, and THz Bands in a 6G CR-SDVN

**DOI:** 10.3390/s25051580

**Published:** 2025-03-04

**Authors:** Umair Riaz, Muhammad Rafid, Huma Ghafoor, Insoo Koo

**Affiliations:** 1 School of Electrical Engineering and Computer Science (SEECS), National University of Sciences and Technology (NUST), Islamabad 44000, Pakistan; uriaz.msee19seecs@seecs.edu.pk (U.R.); mrafid.msee19seecs@seecs.edu.pk (M.R.); 2 Department of Electrical, Electronic and Computer Engineering, University of Ulsan, Ulsan 77024, Republic of Korea

**Keywords:** 6G, cognitive radio networks, dedicated short-range communication (DSRC), millimeter wave (mmWave), software-defined vehicular network (SDVN), terahertz band (THz)

## Abstract

To meet the growing needs of automobile users, and to provide services on demand with stable and efficient paths across different bands amidst this proliferation of users, an integrated approach to the software-defined vehicular network (SDVN) is proposed in this paper. Due to the significant increase in users, DSRC is already considered insufficient to fulfill modern needs. Hence, to enhance network performance and fulfill the growing needs of users in SDVN environments, we implement cognitive technology by integrating the DSRC, mmWave, and THz bands to find stable paths among different nodes. To manage these different technologies, an SDN controller is employed as the main controller (MC), recording the global state of all nodes within the network. Channel sensing is conducted individually for each technology, and sensing results—representing the number of available bands for secondary communications—are updated periodically in the MC. Consequently, the MC manages connections by switching between DSRC, mmWave, and THz bands, providing stable paths between the source and destination. The switching decision is taken by considering both the distance from the MC and the availability of channels among these three technologies. This cognitive integration of bands in SDVN improves performance in terms of network delay, packet delivery, and overhead ratio.

## 1. Introduction

Vehicular ad hoc network (VANET) is a significant component of an intelligent transportation system (ITS), which was purposely developed to control and monitor transportation issues, provide safety for users, reduce traffic accidents, enhance network stability, and many other things. Recently, smart cities have been introduced under the ITS umbrella, where different applications are provided to different users by considering vehicle-to-vehicle (V2V), vehicle-to-infrastructure (V2I), vehicle-to-pedestrian (V2P), vehicle-to-drone (V2D), vehicle-to-ship (V2S), and vehicle-to-everything (V2X) communications [1]. To enhance the performance of these smart cities, various technologies, such as software-defined networking (SDN), artificial intelligence (AI), and network function virtualization (NFV), have been integrated with vehicular networks to gather, handle, and analyze large sets of data for network management. But considering the increasing amounts of vehicular data, the band dedicated to automobile communications—known as Dedicated Short-Range Communication (DSRC), assigned by the FCC in 1997—is no longer sufficient to meet the needs of the growing number of users in a smart city [2]. Among the seven channels of the 75 MHz band, one was solely dedicated to the exchange of control messages, while the remaining six channels, each with a bandwidth of 10 MHz, were exclusively used to exchange data messages. Various cognitive radio (CR) schemes for vehicular environments have been proposed in the literature to overcome spectrum scarcity issues [3,4,5]. But to deal with an upsurge in users (along with services), and to maintain network stability and band availability, a new integrated approach is required.

Spectrum sensing is used to find available bands based on different parameters from the roadside unit (RSU) and to establish stable links between users. To find the optimal band from among the available bands, and to switch frequently between different frequencies, various detection schemes have been used in the literature [6]. Spectrum sensing is a technique that allows vehicles to sense free channels in their current location. Spectrum sensing is a component of CR technology, where secondary users (vehicles) not licensed for a specific band sense the spectrum to determine if primary user activity is not occurring. In order to detect the spectrum, vehicles use an energy detection method, which uses the received power of signals on each channel to select which channels are idle for secondary user communications. Licensed users, called primary users, communicate on the same channel and enable secondary users to use the same band only if their activity is not interrupted. Since spectrum is one of the most expensive and scarce resources, researchers are attempting to utilize it effectively and efficiently to fulfill the increasing demands of all users around the world [7]. Both vehicles and RSUs sense the spectrum and exchange the sensing results to update each other about the availability of bands at specific locations. Hence, cooperation among nodes is required to update the current state. An SDN controller is considered the best entity to manage the locally updated data by recording the global state of the whole network. The controller is also responsible for the decisions to switch between different wireless technologies.

Moving toward 6th-generation (6G) technologies, the services offered by modern vehicles will require ample amounts of bandwidth with high data rates and less delay in the near future [8]. The millimeter wave (mmWave) and terahertz (THz) bands operate within the 30 GHz to 300 GHz range and 0.1 THz to 10 THz range, respectively, with a maximum data rate of 1 Tb/s and extremely low latency of about 0.1 ms, which is over 10 times lower than that of 5G networks. Therefore, we consider these bands as two of the three technologies to meet the current needs for vehicular data. This latest requirement involves high data rates and throughput to properly process the videos and data among vehicular users [9]. Moreover, the integration of vehicular networks in intensive and health applications requires low latency to operate correctly [10]. Similarly, to expedite communications among nano-powered vehicular networks, the THz band plays a vital role in providing stable connections [11]. Consequently, these bands are the best candidates to meet the high-demand vehicle traffic needs.

In terms of data speeds and bandwidth, next-generation wireless technologies perform well with the mmWave band, but both these bands require directional antennas and a direct line of sight (LOS) between the transmitter and receiver to achieve maximum performance. In vehicular environments, these bands face many challenges. To address the problem of high path loss in these high-frequency bands, it is possible to use high-gain antennas and beam-forming with ultra-massive MIMO antennas [12]. Furthermore, the use of the lower THz band can prevent molecular absorption. The distance between the transmitter and the receiver, along with the frequency band, plays a role in maintaining significant attenuation in these technologies. The decrease in network loss is achieved by decreasing both the distance and the frequency band. Accordingly, in addition to the mmWave and THz bands, we also consider DSRC, which is the dedicated spectrum for vehicular communications. Hence, in this proposed scheme, a vehicular node can establish a connection with any of its current neighbors if it finds a free channel among one of these three technologies.

The following are the key contributions of this paper:

(i) A novel scheme for 6G cognitive radio software-defined vehicular networks (CR-SDVNs) is proposed for the city scenario to find stable paths between the source and destination. Communication between two nodes is dependent on the number of available free channels that are provided to each link in forming a stable path. These free channels are sensed by an energy detector scheme that is implemented solely for each wireless technology. Three different technologies (DSRC, the mmWave band, and the THz band) are used to meet the needs of the growing numbers of vehicular users. The SDN controller is responsible for keeping data updated and switching among these three technologies.
(ii) After finding the available channels, the two communicating nodes are allowed to transfer data to each other. Hence, considering the free channels and the link status, the final step is to find an optimal path from among the different available paths between the source and destination. This optimal path can be a combination of different wireless technologies, thereby providing a heterogeneous stable path for modern vehicles in a city environment.

The rest of this paper is organized as follows. Section 2 provides a quick summary and explanation of related work in the literature. Our proposed model and methodology are presented in Section 3, while Section 4 presents the simulation results. Finally, Section 5 concludes the paper.

## 2. Literature Review

Several cognitive schemes integrated with vehicular networks have been proposed in the literature to overcome the problem of spectrum scarcity. Due to an unexpected surge in the number of mobile users, ample amounts of bandwidth with high data rates are urgently needed for these networks to fulfill the growing demands of users. To serve these users, another important factor is the provision of a stable communication path between the source and destination nodes in highly dynamic environments like VANETs. Several solutions to overcome this scarcity problem have been contemplated for a decade, focusing on the stability of the network and providing optimal paths between various sources and destinations [13]. Recently, various emerging technologies, such as the mmWave and THz bands, have been introduced into VANETs to overcome the spectrum scarcity issue and to support the demands of emerging services in VANETs [14,15,16]. In this section, we discuss the contribution of each technology, generally for the 6G and specifically in VANETs.

The concept of integrating these emerging technologies was considered for V2X communications in [17]. The authors considered a linear vehicular network in a small lane, where clusters were formed to facilitate mmWave communications within each cluster, while sub-6 GHz technology was used for inter-cluster communications. A new MAC protocol was proposed to accommodate V2X communications, achieving high throughput and low latency in such an integrated environment. Yi et al. [18] considered both urban and highway scenarios for the THz V2I channel to explore various parameters under different weather conditions. This channel characterization can be used to enable THz-based communications in 6G vehicular networks. Likewise, a multi-band network scheme for vehicular communications was proposed in [19] to ensure LOS transmission in mmWave communicating links. This is a scheduling-based link prediction scheme that considers multiple bands, i.e., DSRC, mmWave, and sub-6 GHz for communications. A hierarchy is first maintained, depending on the distance among vehicles, and then relevant bands of frequency are assigned to the network to achieve higher performance under various conditions.

Another scheme for multicast mmWave vehicular communication was proposed in [20] to achieve high throughput by coordinating communication through sub-6 GHz control messages between relays. The highway scenario was considered to measure the positions of vehicles by using beamwidth-aware scheduling. The results showed that a single transmission can cover up to four receivers. A low THz band that operates in a spectrum of very high frequencies (i.e., between 300 GHz and 1 THz) for vehicular environments in single-lane and multi-lane roadways was proposed in [21] to ensure that communication for this band is dependent on the distance between transmitter and receiver. The results indicated that the THz wave’s trajectory has a significant impact on both penetration and propagation losses. The authors also compared some metrics with mmWave vehicular solutions, and concluded that the THz band outperformed the mmWave. A recent study [22] considered the use of 6G technology to fulfill the demands of next-generation V2X communications. The authors highlighted the use of machine learning based technologies and algorithms specifically designed for efficient vehicular communications. They also discussed the challenges faced by vehicular networks when integrating next-generation vehicular communications.

The protocols discussed above only considered multi-band vehicular communications under different conditions. None of these protocols have been implemented in spectrum-aware vehicular communications by using different wireless bands. In [23], the authors investigated the use of the THz spectrum for automotive communications. With the increase in the world’s population, the number of automobiles is also growing every day, which raises the need for faster data rates and significant bandwidths. The authors discussed the difficulties with THz vehicular communications along with potential solutions. To overcome the issues of bandwidth utilization for vehicular communications, a novel skip network coding (SNC) multipath algorithm was proposed in [24] to reduce the number of packet retransmissions after going through the limitations of traditional multipath techniques. Their simulation results showed significant improvement in terms of packet loss, reliability, and bandwidth utilization. Another energy- and spectrum-aware MAC protocol was proposed to access channels in nano-sensor devices [25]. A distance-based decision was taken for the THz band. To maintain an infinite lifetime, the authors assigned optimal sleeping and transmission times among these devices. Likewise, a novel THz- spectrum-aware scheme named the compression and reconstruction network (CRNet) for vehicular communications was proposed in [26] to improve the accuracy of channel sensing by reducing spectrum complexity. Blind spectrum reconstruction was used in this scheme without considering the locations of vehicles and other channel characteristics. The algorithm demonstrated better results.

The use of cognitive technology for integrating various wireless bands (as mentioned above) has increased the overall delay. This is because finding an optimal channel to make a stable connection between each pair of communicating nodes is a time-consuming procedure. Therefore, a central controller is needed to handle spectrum availability among different wireless technologies and to enable switching between these wireless bands. According to [27], Hu et al. were the first to consider the integration of sub-6 GHz, DSRC, and mmWave bands with mobile edge computing (MEC) in vehicular environments. To distribute larger files among vehicles, MEC and fog computing are used to handle this integration among different bands. The sub-6 GHz band is used for making connections between gateway nodes, whereas mmWave is used for gateway-to-vehicular communications, and DSRC is used for V2V communications. The simulated results demonstrated better performance in comparison to a previous scheme for different network conditions. Likewise, according to [2], the first work that simultaneously considered spectrum sensing and routing for CR-SDVNs was proposed in 2017 to provide stable paths between the source and destination. Routing is a method that involves vehicles choosing links from different neighbors to create a stable route from source to destination. Another CR-SDVN routing scheme was proposed in [28] to enable switching between mmWave and THz bands. Clusters were used to divide the whole network into sub-networks where the THz band was dedicated to communication between vehicles and RSUs. However, for V2V communications, mmWave was used. A fuzzy inference-based system was used to find optimal paths between the source and destination in mmWave communications, whereas in THz communications, the improved fruit fly (IFF) method based on a genetic algorithm (GA) was considered for path selection. The results demonstrated better performance when compared to typical routing for vehicular networks in terms of delivery ratio, delay, and overhead. Ref. [29] proposed a multi-agent algorithm for centralized controllers based on SDN that can improve VANET performance through reinforcement learning. The study utilized a multi-protocol approach and demonstrated superior outcomes across all scenarios.

None of the protocols discussed above simultaneously consider spectrum sensing and routing in a CR-SDVN to identify the number of idle bands available among different wireless technologies. To enhance vehicular communications in the forthcoming 6G, we propose a novel scheme to first find optimal bands that are under-utilized from among different technologies, and then to find the optimal path. For an ITS, the mmWave and THz bands are sufficient to fulfill the needs of the growing number of users by providing high capacity, less delay, and more safety and stability. It is important to sense those under-utilized bands to make them available for vehicular communications. Keeping in mind the pros of these wireless technologies, we integrate the mmWave band with the THz band in the city scenario along with DSRC. Consequently, this is the first work to integrate DSRC, mmWave, and THz bands in a CR-SDVN while simultaneously considering spectrum sensing and routing for both V2V and V2I communications in an urban scenario. Table 1 compares all the schemes set out in this section.

## 3. The Integrated Protocol for a 6G CR-SDVN

We propose a novel cognitive routing protocol that integrates DSRC, mmWave, and THz bands in a 6G SDVN. Our goal is to consider spectrum sensing among these three bands as well as simultaneous routing to find an optimal path between the source and destination in a city scenario. The overutilization of the first band and underutilization of the latter two bands make it necessary for each vehicle to sense the spectrum in order to find the number of available channels for communication. An energy detection scheme is used for each wireless technology individually to sense the spectrum, and then, based on the distances of the communicating nodes from the controller, an optimal path is selected. The main controller (MC) is responsible for maintaining a global record based on the periodic sensing results of all idle bands shared by each vehicle. The MC is also responsible for switching among these bands and providing the optimal path to any querying node.

**Figure 1:**
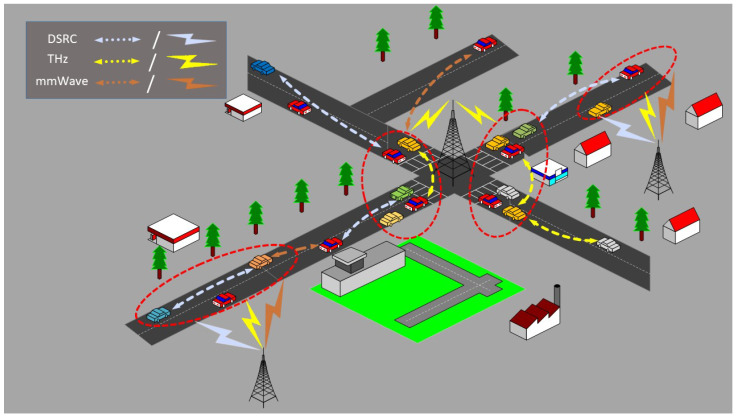
CR-SDVN for DSRC, mmWave, and THz communications in a city scenario.

We consider the integration of DSRC, mmWave, and THz bands in a 6G CR-SDVN with *V* vehicles, *r* RSUs, and an MC, as shown in Figure 1. The MC communicates directly with the RSUs, making it simple for the vehicles to ensure stability. To entice the reader, the MC in the city scenario can be any RSU at an intersection or any traffic police office. All vehicles and RSUs are equipped with three interfaces, one for each wireless technology. Depending on the coverage range of each technology, a vehicle can only form a link with the next relay vehicle moving toward the destination if both have common free channels within that coverage area. This means that the number of channels is assumed to be the same for a specific distance, which in our scheme is the coverage range of each technology. That is, for a specific coverage range, the channel state, which is the presence or absence of a primary user, remains the same, thereby ensuring stability.

A vehicle moving in a city scenario, when looking for a path to a destination, sends a request message (i) to the MC if it is within the coverage range of the vehicle; (ii) to any RSU within the coverage range of the vehicle, which then forwards that request directly to the MC; or (iii) to any neighboring vehicle, which relays the request until it reaches any fixed node. For the above three possibilities, two nodes can only communicate if they have a common free channel. To find these free channels, vehicles need to periodically sense the spectrum while moving, and update the results with each other and with the MC. This sensing is done individually for each band using the energy detector. A dedicated control channel between vehicles and MC is used for this exchange of results. In the next subsection, we explain spectrum sensing for the three different bands in the 6G CR-SDVN.

### 3.1. Spectrum Sensing for DSRC, mmWave, and THz Bands

**Figure 2:**
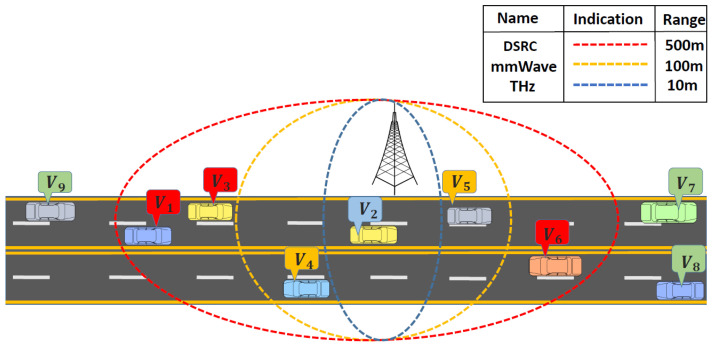
Coverage ranges of DSRC, mmWave, and THz bands.

We consider cognitive technology for different bands to allow exchanges of data with high data rates, low latency, and less overhead among various nodes in vehicular environments. To fulfill these requirements, the MC in our scheme is responsible for ensuring stability by providing optimal bands along with optimal paths to querying nodes. Each vehicle shares sensing results ($SR_{v_{j}}$) with its neighboring nodes up to the coverage range, $C_{i}$, of each band, where *i* represents DSRC, mmWave, or THz, and $v_{j}$ represents vehicle j={1,2,…,V}. The ranges of these bands are shown in Figure 2. These coverage ranges are used to make decisions about switching between different technologies. As mentioned earlier, MC is responsible for switching among these technologies by using a dedicated control channel. $SR_{v_{j}}$ is updated periodically by each vehicle, and the list of free channels obtained by each vehicle, $LFC_{v_{j}}$, is maintained at the global level by the MC as shown in Table 2.

Thus, each vehicle updates its neighbors with the following entries to find $LFC_{v_{j}}$:

(1) $$SR_{v_{j}}=<C_{i},f_{i},E_{C_{i},f_{i}},\eta_{C_{i},f_{i}}>$$

where $f_{i}$ represents one of the $M_{i}$ channels ($M_{i}$ is the total number of channels in wireless technology *i*), $E_{C_{i},f_{i}}$ represents the energy of the signal on channel $f_{i}$ within the coverage range $C_{i}$, and $\eta_{C_{i},f_{i}}$ is the number of samples.

To explain how vehicles exchange sensing results with each other, let us consider the example scenario in Figure 2. As seen in the figure, $V_{1}$ (within the DSRC range) wants to communicate with $V_{2}$ within the THz range. The former senses the spectrum by using the energy detector equations:

(2) $$H_{z}{\left(C_{i},f_{i}\right)}_{V_{j}}=\left\{\begin{array}{cc} 0, & \mathrm{if}\;E_{C_{i},f_{i}}^{V_{j}}\leq \rho \\ 1, & \mathrm{if}\;E_{C_{i},f_{i}}^{V_{j}}\;>\rho \end{array}\right.$$

ρ represents the threshold. Equation (2) indicates that there are always two possibilities: either a channel is present within the communication range or not.

(3) $$E_{C_{i},f_{i}}^{V_{j}}=\left(1-\alpha \right)E_{C_{i},f_{i}}^{V_{j}}+\alpha E_{C_{i},f_{i}}^{V_{j+1}}$$

(4) $$\alpha =\frac{\eta_{C_{i},f_{i}}^{V_{j+1}}}{\eta_{C_{i},f_{i}}^{V_{j}}+\eta_{C_{i},f_{i}}^{V_{j+1}}}$$

Equation (3) explains a merging rule, where two moving vehicles that wish to connect, depending on the channel’s availability, exchange their sensing results. However, α in (4) represents the ratio between the number of sensing samples in $V_{2}$ and the sum of sensing samples between two vehicles. All available channels for $V_{1}$ are shown as $LFC_{V_{1}}$ = $H_{0}\left(C_{DSRC},f_{DSRC}\right)$. Likewise, all available channels for $V_{2}$ are $LFC_{V_{2}}$ = $H_{0}\left(C_{i},f_{i}\right)$. The common channels between both $V_{1}$ and $V_{2}$ must be in the DSRC band (because $V_{2}$ is within 500 m range of $V_{1}$), so these vehicles exchange data using one of the common $M_{DSRC}$ channels. This is an example scenario showing a direct link between $V_{1}$ and $V_{2}$ only. If no common DSRC channel is available for this link, then $V_{1}$ must select $V_{3}$ within a 10 m distance at the THz band to reach $V_{2}$. Keeping the global record of coverage ranges helps other vehicles find available channels for specific locations. When $V_{9}$ reaches the current position of $V_{1}$, it can request the list of free channels from the MC at that position if it is within range of the MC. Consequently, coordinating sensing results among vehicles and managing the globally updated state through the MC increases path stability in such a dynamic environment, which will be discussed in the next subsection.

### 3.2. Path Selection Between the Source and Destination for Three Different Bands

This section illustrates how a data packet is transferred from any source node to the destination node in a city scenario using $LFC_{v_{j}}$ for three different bands. From our previous discussion, it is clear that the coverage range is the basic parameter to reach a final decision in the available spectrum. Vehicles exchange control messages with each other or with the MC by using a control channel. As a result of this information exchange, each vehicle has the following entries in its flow table:

(5) $$<V_{j},V_{x,y},V_{speed},LFC_{V_{j}}>$$

These entries are used among neighbors to form a stable link between two moving vehicles. When source node *j* wants to send data to any destination node *k* that is not within communication range, it sends a request message (packet_in) to the MC. For any source node, there are two possibilities: (i) the MC is within the source’s coverage range, and (ii) the MC is not within range. In the former case, the MC replies to the source node with a route to the destination as follows:

(6) $$route_{v_{jk}}=max\left(PD_{p}\right)$$

where $PD_{p}$ is the path duration for all available paths between the source and destination, and

(7) $$PD_{p}=min\left(LD_{1,p},LD_{2,p},\ldots ,LD_{l,p}\right)$$

where p={1,2,…,P} is the total number of available paths between the source and destination, *l* is the total number of links for each path between the source and destination, and LD is the link duration, which is calculated as follows:

(8) $$LD_{jk}=\frac{C_{i}\pm d_{jk}+CC_{jk}}{\sqrt{{\left(s_{j}\cos\theta_{j}-s_{k}\cos\theta_{k}\right)}^{2}+{\left(s_{j}\sin\theta_{j}-s_{k}\sin\theta_{k}\right)}^{2}}}$$

where $C_{i}$ represents the coverage range of each band, *s* is the speed, θ is the angle, $CC_{jk}=\left\{LFC_{v_{j}}\cap LFC_{v_{k}}\right\}$, ± indicates both directions, and the distance $d_{jk}=\sqrt{{\left(x_{j}-x_{k}\right)}^{2}+{\left(y_{j}-y_{k}\right)}^{2}}$. This (8) monitors how long two vehicles remain connected to each other. For the latter case, the source finds the relay node by using (8) to reach the MC. This relay node could be any other vehicle within the source’s coverage or that of a nearby RSU. With the RSU, the delay from the RSU to the MC is considered negligible because both are directly connected in our proposed scheme.

Now, the question is how these vehicles decide which band they are going to communicate with and ensure stability. The three wireless technologies we use in this proposed scheme have different frequency ranges, bandwidths, data rates, coverage areas, and underutilized spectrum. In this paper, we consider the coverage range for the DSRC band to be 500 m, for the mmWave band 100 m, and for the THz band 10 m, as seen in Figure 2. These ranges can vary from environment to environment. We assume clear weather in this scheme. We will analyze the environmental aspects associated with each of these technologies in the near future. In order to explain the decision-making process over the communication band (including common free channels, switching between these bands, and ensuring a stable path via the MC), we considered five different cases in the city model with the help of an example scenario.

**Figure 3:**
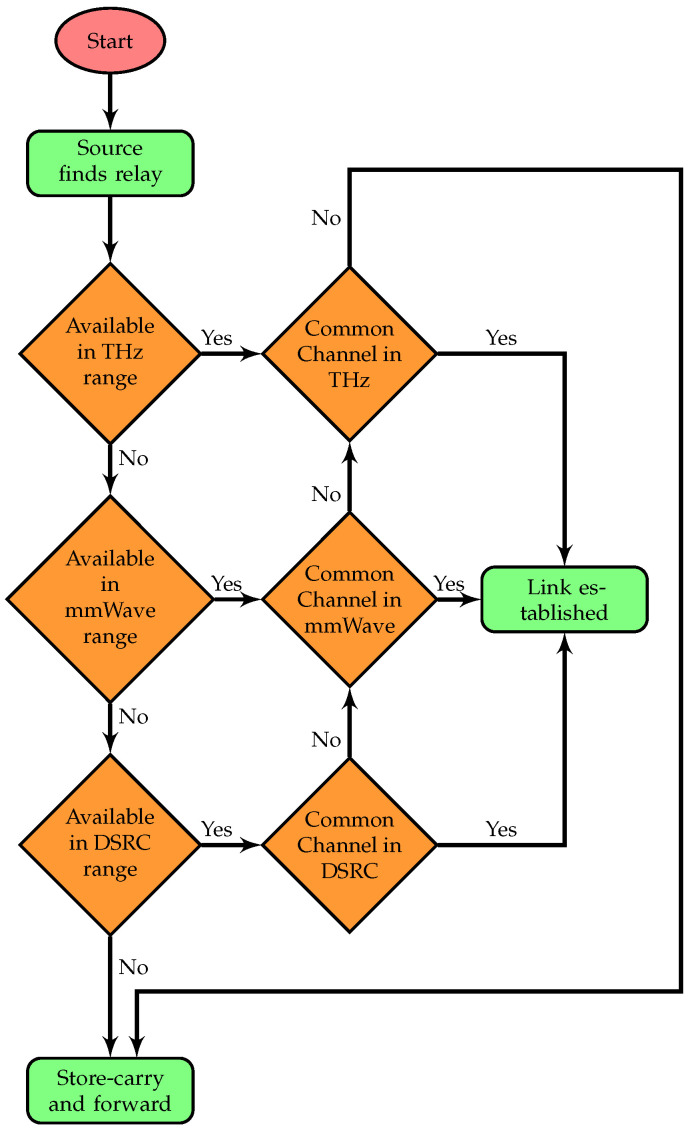
A flowchart representing the five cases in a 6G CR-SDVN.

- *Case i: (when the source vehicle is within the DSRC range)* After exchanging sensing results with neighboring vehicles and the MC using a control channel, all nodes that act as relay nodes for the source store that information in their flow tables and update it periodically. Hence, whenever a source node sends the request message to the MC, the MC calculates the node’s distance to its neighboring nodes based on the current position of the vehicle. If the distance is less than 500 m and both nodes have common free channels in the DSRC band, the MC selects this node as the first relay. It then repeats this process for the second relay and does so until it reaches the destination. For each link, based on the current position of the two communicating nodes and the range in which these nodes lie, these nodes only make a stable link for that specific band. In this way, a path between the source and destination can be the combination of three different wireless technologies.
- *Case ii: (when the source vehicle is within the DSRC range but no free common channels are available)* If there is no common channel available in the DSRC band in the previous case, which is highly possible in any vehicular environment, the vehicle checks the other bands. As mentioned above, the channel state remains the same for a specific coverage range. Since 500 m covers both mmWave and THz ranges, there is a possibility that the querying vehicle may find common free channels in another band, but it can select a relay only within the coverage range of the respective band. In order to forward the packet without delay, if the source and relay nodes are 100 m or 10 m apart from each other, and both have the same free channel for either distance, they will form a link. If a source node discovers that both mmWave and THz bands are available, it will choose to use the mmWave band to send the packet to the furthest distance. To elaborate on this, refer to Figure 2, where the following list could be possible for a $V_{1}$ to $V_{3}$ link. $LFC_{V_{1}}$ = $H_{0}\left(C_{DSRC,mmWave,THz},f_{DSRC,mmWave,THz}\right)$ and $LFC_{V_{3}}$ = $H_{0}\left(C_{DSRC,mmWave,THz},f_{DSRC,mmWave,THz}\right)$.
- *Case iii: (when the source vehicle is within mmWave range)* In this case, we consider all the relay nodes to be within the mmWave range only. After calculating LD, which includes free channels in the mmWave range, the MC repeats the process until it reaches the destination, and finally sends the heterogeneous stable path to the source vehicle. From our example scenario in Figure 2, $V_{1}$ and $V_{4}$ can make a possible connection for this case if both have the following lists available: $LFC_{V_{1}}$ = $H_{0}\left(C_{DSRC,mmWave},f_{DSRC,mmWave}\right)$ and $LFC_{V_{4}}$ = $H_{0}\left(C_{DSRC,mmWave},f_{DSRC,mmWave}\right)$.
- *Case iv: (when the source vehicle is within mmWave range but no common channel is available)* If no common channel is available for source and relay nodes in the mmWave range, the source vehicle checks the THz band. Since the THz range covers 10 m, there is a possibility that the querying vehicle has common free channels in this band. To ensure delivery, if the source and relay nodes are 10 m away from each other, and both have the same free channel in the THz band, they form a link. As discussed in Caseii, the same link from $V_{1}$ to $V_{3}$ for THz communication is used to deliver the packet in this scenario.
- *Case v: (when the source vehicle is within the THz range)* In this case, there are two possibilities: (i) when the relay node is only 10 m away from the source vehicle and all neighboring vehicles are within this range, it means the vehicle must be at an intersection; at an intersection, the source must be within range of an RSU, so the querying vehicle repeats the previous four cases (to find $CC_{jk}$) to make a stable connection between itself and the RSU; and (ii) when a source node does not have a node within 500 m, except for the one that is just 10 m away from the source. This relay might have another node that is 510 m from the source. In all cases, if the source vehicle fails to find a free common channel or a relay node, the vehicle then holds the packet under the store–carry–forward scheme until it finds a free channel or a relay node. A delay might occur by using this approach, but the delay is more acceptable than dropping the packet. To summarize, using three different wireless technologies, the scheme allows a packet to be forwarded to the farthest node if the channel for that technology is available. Otherwise, if the spectrum is not found in one band, there is a good chance that vehicles can still establish a stable link by using one of the other available bands within the coverage range. The flow chart summarizing the whole algorithm is shown in Figure 3.

## 4. Simulation Results

This section presents an evaluation of the proposed scheme in terms of different quality of service (QoS) parameters.

### 4.1. Simulation Environment

The simulation model for the proposed scheme was created by integrating two different environments. The open-source simulation platform NS-2 was used to model and analyze the proposed topology. Since the topology only considers the city scenario, the SUMO traffic simulator was used to model the mobility of vehicles in it. SUMO is also an open-source traffic simulation tool that is used to create vast networks for both highway and city scenarios. We used the city scenario to test the proposed idea with low-speed vehicles. With intersections in the city scenario (unlike vehicles in a highway scenario), it is quite simple to handle stability in a dynamic environment. Moreover, the coverage range of the THz band ensures stability when there is no other relay node, except for one within the THz range. We will consider the highway model in future work. The route file created from SUMO can be reused with NS-2 and many other simulation tools. The underlying protocol, OpenFlow, was chosen because it is widely accepted and customizable when it comes to routing strategies. The message-passing mechanism in NS-2 underwent modification to match OpenFlow message exchanges. The software versions used included NS-2.35 and SUMO-1.14.1.

**Figure 4:**
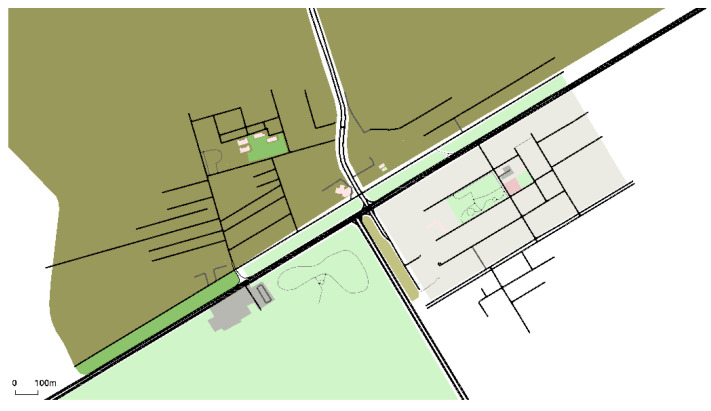
Our mobility model’s simulation environment.

The mobility model for vehicles can be seen in Figure 4. We considered a total area of 1500 m × 3000 m. The number of RSUs was set at r=3, and the numbers of DSRC, mmWave, and THz channels, denoted as $M_{i}$, were 5, 25, and 27, respectively, with coverage ranges, $C_{i}$, of 500 m, 100 m, and 10 m. A dedicated 10 MHz band was designated as a common control channel. Simulations were performed for 25, 50, 75, and 100 vehicles, each with an average speed of 20 m/s, which varied between 10 m/s and 25 m/s for different simulation times (100 s and 150 s). We contemplated using the Nakagami distribution in the DSRC scenario to handle both small- and large-scale propagation effects. We chose the Nakagami model for the radio channel characteristics of DSRC because it is widely accepted for describing the statistical characteristics of both small-scale and large-scale fading. The other two bands have many challenges in vehicular environments when it comes to these technologies. As discussed in Section 1, utilizing high-gain antennas and beam-forming with ultra-massive MIMO antennas can solve the high path loss problem in these high-frequency bands. Additionally, by opting for a lower THz band, we can prevent molecular absorption. Both the distance between the transmitter and receiver, as well as the frequency, impact the high attenuation of these technologies. Reducing both the distance and frequency bands results in decreased network loss. Optimizing the distance and frequency for these technologies has enabled us to achieve reduced path loss, as demonstrated by our simulation results, resulting in less performance degradation across all three parameters. We utilized the same path loss and propagation models outlined in [12]. Moreover, the statistical channel model of NS-2 was employed because it depends on the 3GPP channel model in the band between 6 and 100 GHz. Table 3 shows important simulation parameters in tabular form.

This is the first work considering simultaneous spectrum sensing and routing for three different wireless technologies. Therefore, as mentioned in Section 1 and Section 2, no similar protocol is available for comparison. Hence, we considered reference schemes implemented for each technology individually in order to compare our proposed scheme (reference scheme with DSRC [17], reference scheme with mmWave [19], and reference scheme with THz [28]). To make a fair comparison, we implemented the scheme from [19] by integrating SDN and CR technology, and considered a cluster to be the mmWave coverage range only for this reference scheme. For the THz reference scheme, we only simulated the band by considering the idea from the authors of [28]. The same simulation environment and parameters were considered to simulate all reference schemes. Simulations were conducted to evaluate the performance of our proposed scheme by using the following three metrics:

(i) Packet delivery ratio (PDR): This is the ratio of packets successfully received by the destination to the total number of packets sent from the source. If $sent_{n}$ denotes the number of packets sent and $rcvd_{n}$ represents the number of successfully received packets, then PDR is calculated as follows:
  (9) $$PDR=\sum_{n=1}^{m}\left(\frac{rcvd_{n}}{sent_{n}}\right)*100$$
(ii) End-to-end delay: This is the total time required by a packet to travel from the source to destination, calculated as follows:
  (10) Delay=packetarrivaltime−packetdeparturetime
(iii) Routing overhead ratio (ROR): This is the ratio of the number of control packets to the total number of packets in the network. If $cntrl_{n}$ denotes the number of control packets sent, and $total_{n}$ denotes the total number of packets, the overhead is calculated as follows:
  (11) $$ROR=\sum_{n=1}^{m}\frac{cntrl_{n}}{total_{n}}$$

### 4.2. Results Analysis

A summary of our results based on comparisons of the three individual wireless technologies for the above three metrics is presented in Table 4. In this section, we discuss the results for each parameter in detail.

#### 4.2.1. Packet Delivery Ratio

**Figure 5:**
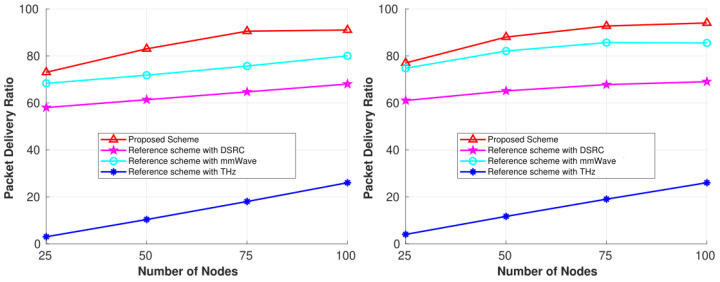
Performance comparisons for the 6G CR-SDVN in terms of PDR at 100 s and 150 s.

Figure 5 shows the PDR comparison between our proposed scheme and the three individual reference schemes (DSRC, mmWave, and THz) in the city scenario for two different simulation times. PDR increased with increases in the number of nodes in the network as well as with the increase in simulation time. This proved our objective of maintaining stability in the network for such a highly dynamic and heterogeneous network. Increasing the number of nodes and the simulation time improved network connectivity; if free bands were available, vehicles could form stable links for longer durations. The three communication technologies in this highly dynamic network enable free bands to be available, which improves the performance of our proposed scheme, as stable links depend on the availability of idle channels. We can see from Figure 5 that the proposed scheme achieved higher PDR than the reference schemes. The reason is simple. With three different technologies in the network, the proposed scheme increases the number of common free bands. Based on the coverage distance between nodes, if the spectrum is not found in one band, there is a good chance that vehicles can still establish a stable link by using one of the other available bands within the coverage range. As vehicles periodically sense the spectrum and update the MC with their current state, the MC maintains the network’s global state, which helps any vehicle that joins the network at some later time to keep a stable connection with a destination node, as discussed in the previous section. Thus, both figures demonstrate that by calculating the shortest stable path—considering both link duration and the number of common free channels across three different wireless technologies simultaneously—our scheme outperforms the other three.

The THz reference scheme showed a poor delivery ratio compared to the mmWave and DSRC reference schemes. This is due to the smaller coverage area for the THz band. Ideally, this PDR should be higher than other bands because the spectrum has a large number of unused bands, but the small coverage range of approximately 10 m degrades the overall performance in vehicular networks. For moving vehicles, it is unusual to maintain a stable connection for just a 10 m distance. It is only possible for this band to maintain stability at intersections, where its benefits are obtained in our proposed scheme, as can be seen in all simulation results. For the other two schemes, DSRC has a large coverage range but the spectrum is too small to fulfill all the demands of vehicular users individually. This limited spectrum of about 75 MHz makes the performance poor by dropping packets after not finding a free common channel. But if bands are available, the scheme can outperform the other reference schemes because it has a large coverage range. Likewise, the mmWave reference scheme demonstrated better performance than both the DSRC and THz schemes. Because the coverage range is 10 times better than the THz band, but is less than the DSRC band, maintaining a stable connection for only a 100 m range is difficult in vehicular networks. Because the spectrum has a large number of free bands, in comparison to DSRC, it outperformed both reference schemes. Our proposed scheme achieved a maximum delivery of 94%.

#### 4.2.2. End-to-End Delay

**Figure 6:**
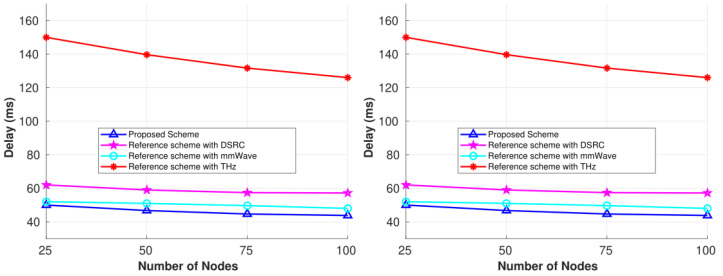
Performance comparisons for the 6G CR-SDVN in terms of end-to-end delay at 100 s and 150 s.

Figure 6 shows the comparison of end-to-end delay from our proposed scheme and the individual reference schemes for each wireless technology in the city scenario for the two simulation times. From the figure, we can see that increasing the time window improves network performance because vehicles have more time to establish stable connections. The overall delay for all the schemes showed the same pattern, i.e., increasing the number of vehicles decreases the delay. With fewer vehicles, it is more difficult to maintain stability by finding both a relay and a common free channel simultaneously. For the individual schemes, if a relay is found for a stable connection, it is possible that a common free channel is not available, so no connection is established, and the network incurs a delay in transmitting the packet successfully. However, in our case, we have three different bands available; from among those three bands, there is a good chance that a common free channel is available for the specific coverage range, thereby improving network performance. Because the MC is responsible for recording the global state, in such a scenario, the MC provides a stable heterogeneous path that might switch between different bands. A vehicle attempting to connect through DSRC can switch to the mmWave or THz band if DSRC is not available at that time. Moreover, in a sparse network, if a condition occurs where a vehicle does not find a relay and a channel under our scheme, it keeps the packet and forwards it as soon as it reaches a node within the coverage range. In sparse conditions, Figure 6 shows that there is a slightly higher delay because there are not enough available vehicles. Packets can be stored for longer periods of time before being delivered to the next available node when using any of the three wireless technologies. Considering three different technologies increases the likelihood of a vehicle reaching the coverage range of an RSU or a relay sooner; this is because we have 10 m, 100 m, and 500 m ranges available in our proposed scheme, demonstrating improvement compared to the individual schemes.

However, for each reference scheme, when a vehicle does not find a relay node or a common free channel (or both), it will drop the packet, which degrades network performance. Because the coverage range of the THz band is smaller, the THz reference scheme shows high end-to-end delay compared to the DSRC and mmWave schemes. The reason is the same as mentioned for PDR, i.e., due to the smaller coverage range, it is more difficult for vehicles to maintain a connection, and thus, the packet drop rate is high in this band, even though the number of free channels is high. It takes time for a vehicle to reach a destination if a 10 m coverage range is used to maintain connections, which is unusual and not possible in vehicular networks. Likewise, the DSRC reference scheme has a high coverage range, but the free bands are congested. We benefit from this coverage range in our proposed scheme by using free available mmWave or THz bands since both coverage ranges lie within 500 m, thereby maintaining stability. Because the mmWave band acts as the median band based on the coverage range between DSRC and the THz band, and it has a large number of unused bands, it demonstrated better performance in comparison to the DSRC and THz reference schemes. We achieved a minimum end-to-end delay of 43.8 ms and a maximum of 50.3 ms under our proposed scheme. For the reference schemes, the minimum values were 48 ms, 57.2 ms, and 126 ms with maximum values of 52 ms, 62 ms, and 150 ms for the mmWave, DSRC, and THz bands, respectively.

#### 4.2.3. Routing Overhead Ratio

**Figure 7:**
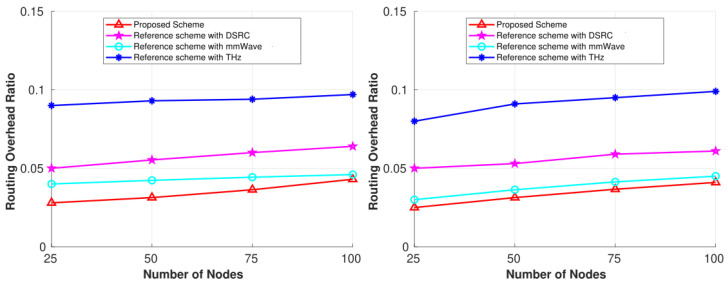
Performance comparisons for the 6G CR-SDVN in terms of ROR at 100 s and 150 s.

Figure 7 compares the ROR for our proposed scheme and the individual reference schemes (DSRC, mmWave, and THz) in the city scenario for the same two simulation times. From the figure, we can see that ROR increases with an increase in network density and the time window. To establish a link in any type of communication network, a node needs to exchange control packets to share the local state with its neighboring nodes. In our scheme, a vehicle needs to periodically update the MC as well as neighboring vehicles with channel sensing results and other parameters required for routing. This is done by exchanging control packets among nodes. The reason the proposed scheme outperformed the three reference schemes is that it updates the MC periodically about its local state. The global state kept by the MC helps any querying vehicle to find a stable path with fewer query messages (i.e., control messages in conventional routing schemes), thereby reducing the overall number of control packets in the network. Another reason is that since the proposed scheme integrates three different technologies, the network has a lower chance of failure when searching for a relay node with a common free channel, as switching is provided by the MC. Due to this low possibility, the number of control messages exchanged is low, reducing the network burden. As discussed above, the THz reference scheme shows a large overhead in comparison to the DSRC and mmWave schemes. The reason is due to the large number of exchange messages required when links among vehicles break frequently due to a smaller coverage area. Therefore, vehicles make several attempts to send control packets periodically in order to connect to the network and build pathways, causing the overhead ratio to rise. The DSRC reference scheme showed improvement in comparison to the THz scheme, but it has a large overhead when compared with the mmWave scheme. For DSRC, when the free channel is not available, the links break frequently in the same way as the THz scheme, but the large coverage range of DSRC outperforms the THz scheme. Among the reference schemes, the mmWave band demonstrated the second-best performance in terms of overhead ratio. A good coverage area with a sufficient number of channels results in fewer control packet exchanges compared to the DSRC and THz schemes. For all the schemes considered in this paper, there is a clear trend of the overhead ratio increasing as the number of vehicles rises. But we achieved an optimal value by including the controller, as shown for the proposed scheme in Figure 7. Consequently, our simulation results demonstrate that our proposed scheme performs better than the three reference schemes. The integration allows us to achieve better performance when channels are not available in one wireless technology or when vehicles are unavailable to serve as relays. Our scheme allows us to switch to other available technologies that have idle bands among the three technologies and maximize the delivery of packets to the destination with less delay and overhead. Also, these results demonstrate that integrating different technologies and considering spectrum sensing and routing can simultaneously increase network performance, even for such a highly dynamic network.

## 5. Conclusions

In this paper, we introduced a novel 6G cognitive radio software-defined vehicular network. The idea is to integrate different wireless technologies by combining spectrum sensing, switching, and routing to provide stable paths between the source and destination vehicles. The common free channels from among the DSRC, mmWave, and THz bands, sensed by each moving vehicle using an energy detector method, are considered between communicating vehicles to establish stable links. A main controller is responsible for recording the global state of the network, and it provides stable heterogeneous paths on demand to any querying nodes. These routing paths are selected by considering the common channels, speed, distance, and coverage range of each wireless technology. The controller is also responsible for managing switching between bands. By considering the coverage range and the availability of common free channels at a specific location, switching decisions are taken. Therefore, based on the availability of channels, a path between any source and destination can be a combination of different wireless bands. Our simulation results show better performance in terms of packet delivery ratio, end-to-end delay, and overhead ratio. We plan to sub-layer the SDN control layer in the near future, allowing different controllers at global and local levels to manage network message exchanges in our proposed scheme. Future work will also take into account both highway and city scenarios to assess the performance of both 5G and 6G technology bands by employing CR technology in vehicular networks. Furthermore, we are planning to extend this work to autonomous vehicles as well.

## Figures and Tables

**Table 1:** Comparison of related schemes.

References	Consider City Environment?	Consider the Spectrum Scarcity Issue?	Consider Stable Routing Paths?	Consider SDN?	Consider Consider Different Wireless Technologies?			
					DSRC	mmWave	THz	Integration of Three
[17]	✗	✗	✗	✗	✓	✓	✗	✗
[18]	✓	✗	✗	✗	✗	✗	✓	✗
[19]	✗	✗	✗	✗	✓	✓	✗	✗
[20]	✗	✗	✗	✗	✗	✓	✗	✗
[21]	✗	✗	✗	✗	✗	✓	✓	✗
[22]	✗	✗	✗	✗	✓	✓	✓	✗
[23]	✗	✗	✗	✗	✗	✗	✓	✗
[24]	✗	✗	✓	✗	✗	✗	✗	✗
[25]	✗	✓	✗	✗	✗	✗	✓	✗
[26]	✗	✓	✗	✗	✗	✗	✓	✗
[27]	✗	✗	✓	✓	✓	✓	✗	✗
[28]	✗	✓	✓	✓	✗	✓	✓	✗
[29]	✗	✗	✓	✓	✓	✗	✗	✗

**Table 2:** MC information table.

Vehicle ID	Vehicle Position	Vehicle Speed (m/s)	${\mathrm{LFC}}_{v_{j}}$		
$V_{1}$	$\left(x_{1},y_{1}\right)$	25	$Ch_{1,DSRC}$ $Ch_{3,DSRC}$ $Ch_{5,DSRC}$	$Ch_{6,mmWave}$ $Ch_{9,mmWave}$	$Ch_{3,THz}$ $Ch_{2,THz}$
$V_{2}$	$\left(x_{2},y_{2}\right)$	20	$Ch_{5,DSRC}$	$Ch_{8,mmWave}$	$Ch_{1,THz}$
⋮	⋮	⋮	⋮	⋮	⋮
⋮	⋮	⋮	⋮	⋮	⋮
⋮	⋮	⋮	⋮	⋮	⋮
$V_{V}$	$\left(x_{V},y_{V}\right)$	30	$Ch_{1,DSRC}$	$Ch_{6,mmWave}$ $Ch_{9,mmWave}$ $Ch_{8,mmWave}$	$Ch_{3,THz}$ $Ch_{1,THz}$ $Ch_{5,THz}$

**Table 3:** Simulation parameters.

Parameters		Values
Area		1500 m × 3000 m
Velocity		10–25 m/s
Packet Size		64 bytes
Number of RSUs		3
Simulation Time		100 s and 150 s
Number of vehicles		25, 50, 75, 100
DSRC	Coverage Range	500 m
	Freq Range	5850–5925 MHz
	Bandwidth	10 MHz
	Antenna Gain	10 dBi
	Tx power	27 dBm
mmWave	Coverage Range	100 m
	Freq Range	30–60 GHz
	Bandwidth	30 GHz
	Antenna Gain	17 dBi
	Tx power	20 dBm
THz	Coverage Range	10 m
	Freq Range	0.3–3 THz
	Bandwidth	0.1 THz
	Antenna Gain	24 dBi
	Tx power	10 dBm

**Table 4:** Comparison of the bands for PDR, end-to-end delay, and ROR.

Spectrum Band	Drawbacks
DSRC	The PDR value is low, with a high end-to-end delay and overhead ratio. This shows a scarcity of the dedicated spectrum for vehicular communications.
mmWave	All three parameters show quite good performance in comparison to both DSRC and THz bands. This is due to a good number of unused bands and a good coverage area.
THz	All parameters show poor results because of the low coverage range.

## Data Availability

Data are contained within the article.

## Abbreviations

The abbreviations employed in this article are displayed below.

DSRC	dedicated short-range communication
mmWave	millimeter wave
THz	terahertz
6G	sixth generation
SDN	software-defined networking
CR-SDVN	cognitive radio–software-defined vehicular network
MC	main controller
VANET	vehicular ad hoc network
ITS	intelligent transportation system
V2V	vehicle-to-vehicle
V2I	vehicle-to-infrastructure
V2P	vehicle-to-pedestrian
V2D	vehicle-to-drone
V2S	vehicle-to-ship
V2X	vehicle-to-everything
AI	artificial intelligence
NFV	network function virtualization
FCC	Federal Communications Commission
RSU	roadside unit
LOS	line of sight
NS-2	network simulator-2
PDR	packet delivery ratio
ROR	routing overhead ratio
